# Intestinal Microbiota Disruption Reduces Regulatory T Cells and Increases Respiratory Viral Infection Mortality Through Increased IFNγ Production

**DOI:** 10.3389/fimmu.2018.01587

**Published:** 2018-07-10

**Authors:** Mitchell H. Grayson, Lauren E. Camarda, Syed-Rehan A. Hussain, Sarah J. Zemple, Michael Hayward, Vy Lam, Desiré A. Hunter, Jennifer L. Santoro, Michelle Rohlfing, Dorothy S. Cheung, Nita H. Salzman

**Affiliations:** ^1^Division of Allergy and Clinical Immunology, Medical College of Wisconsin, Milwaukee, WI, United States; ^2^Division of Allergy and Immunology, Nationwide Children’s Hospital and The Ohio State University, Columbus, OH, United States; ^3^Division of Pulmonary and Sleep Medicine, Medical College of Wisconsin, Milwaukee, WI, United States; ^4^Division of Gastroenterology, Department of Pediatrics, Medical College of Wisconsin, Milwaukee, WI, United States

**Keywords:** virus, respiratory infection, gastrointestinal microbiome, pulmonary immune response, pulmonary microbiome, innate lymphoid cells

## Abstract

Alterations in gastrointestinal microbiota indirectly modulate the risk of atopic disease, but effects on respiratory viral infections are less clear. Using the murine paramyxoviral virus type 1, Sendai virus (SeV), we examined the effect of altering gastrointestinal microbiota on the pulmonary antiviral immune response. C57BL6 mice were treated with streptomycin before or during infection with SeV and resulting immune response studied. Ingestion of the non-absorbable antibiotic streptomycin led to a marked reduction in intestinal microbial diversity without a significant effect on lung microbiota. Reduction in diversity in the gastrointestinal tract was followed by greatly increased mortality to respiratory viral infection (*p* < 0.0001). This increase in mortality was associated with a dysregulated immune response characterized by decreased lung (*p* = 0.01) and intestinal (*p* = 0.03) regulatory T cells (Tregs), and increased lung IFNγ (*p* = 0.049), IL-6 (*p* = 0.015), and CCL2 (*p* = 0.037). Adoptive transfer of Treg cells or neutralization of IFNγ prevented increased mortality. Furthermore, Lin^−^CD4^+^ cells appeared to be a potential source of the increased IFNγ. Together, these results demonstrate gastrointestinal microbiota modulate immune responses at distant mucosal sites and have the ability to significantly impact mortality in response to a respiratory viral infection.

## Introduction

The intestinal microbiome plays essential roles in host physiology, nutrition, immune development, and protection (1). Specific members of this complex ecosystem have been implicated in the development of both pro-inflammatory (2–6) and regulatory (3, 7–9) immune responses. In genetically susceptible mice, there is evidence that the intestinal microbiota drive development of colitis (7, 9, 10), type 1 diabetes (11), obesity (12), autoimmune arthritis (6), and experimental autoimmune encephalomyelitis (5). The mechanisms by which the microbiota influence these disease processes are complex and not entirely clear. In humans, the microbiota are directly associated with diseases such as inflammatory bowel disease and necrotizing enterocolitis (13, 14). More indirect relationship studies have suggested that the intestinal microbiota may play a role in modulating the risk for many other clinical diseases including asthma and allergic disease (15–18).

Antibiotic use causes significant and persistent disruption of the intestinal microbial ecosystem (19, 20). This disruption has been associated with increased susceptibility to intestinal infection. In mice, pretreatment with antibiotics is a common approach used to study infection by diverse enteric pathogens, including *Salmonella enterica* (21), *Enterococcus faecalis* (22), and *E. coli* (23). These observations support the concept that an intact microbiota provides colonization resistance to enteric pathogens. In humans, there is similar evidence of antibiotic-associated intestinal infections, such as *Clostridium difficile* (24, 25) and *E. faecalis* (26), normally held in check by an intact microbiota, and successfully treated by reconstitution of the intestinal microbiota with fecal transplantation (27, 28). While it is clear that antibiotic modulation of the intestinal microbial ecosystem alters the immune system in the gastrointestinal tract, evidence suggests that disrupting the intestinal microbiota alters immune responses at distant mucosal and non-mucosal sites (5, 6, 29, 30).

One distant mucosal site to the intestine is the respiratory tract. We undertook this study to see how alterations in the intestinal microbiota might affect the immune response to a natural rodent respiratory viral pathogen, Sendai virus (SeV). The mouse parainfluenza virus type 1, SeV, is a rodent pathogen of the *paramyxoviridae* family [same family as respiratory syncytial virus (RSV) and human parainfluenza viruses 1–3]. Our studies and others have well characterized the antiviral immune response to SeV (31–34). C57BL6 mice infected with the appropriate inoculum lose nearly 20% of their body weight over 10 days, and clear the virus around day 10–12 post inoculation (PI). Fewer than 5% of infected wild-type mice die of the infection, and those that survive are left with mucous cell metaplasia that is dependent on IgE and the high-affinity receptor for IgE, FcεRI, on lung dendritic cells (31, 33). Using SeV, we sought to determine if alteration of the intestinal microbiota by a non-absorbed antibiotic would lead to changes in the respiratory immune response against a well-characterized respiratory viral infection.

## Materials and Methods

### Mouse Handling and SeV Inoculation

C57BL/6, *Cd8*^−^*^/^*^−^, and *Foxp3^gfp^* male mice were obtained from The Jackson Laboratory (Bar Harbor, ME, USA) at 6 weeks of age. Upon arrival at our facility, mice were given either reverse osmosis (RO) drinking water or RO water supplemented with 0.5 g streptomycin sulfate (MP Biomedicals, Solon, OH, USA) per 250 mL RO water and allowed to drink *ad libitum* for various durations depending on the experimental design depicted in section “Results.” Water bottles containing streptomycin were replaced twice weekly. For SeV inoculation, mice were sedated with a ketamine xylazine mixture and inoculated intranasally with 30 µL of 2 × 10^5^ plaque forming units SeV (Fushimi strain; American Type Culture Collection, Manassas, VA, USA) or UV inactivated SeV (UV-SeV), as we have previously described (31). All experiments were approved by the Institutional Animal Care and Use Committee.

### Real-Time Polymerase Chain Reaction (PCR), Flow Cytometry, and Cytokine Microarray

Real-time PCR assays were performed as we have previously reported (31, 32). Messenger RNA was isolated from whole lungs of mice with TRIzol (Invitrogen, Carlsbad, CA, USA). Complementary DNA was made using Quanti-Tect Reverse Transcription kit (Qiagen, Valencia, CA, USA). Real-time PCR assays were performed on the StepOnePlus PCR system using TaqMan Fast Universal PCR master mix (Applied Biosystems, Carlsbad, CA, USA). SeV primer and probes were a gift from Michael J. Holtzman and Eugene Agapov (Washington University, St. Louis, MO, USA). TaqMan gene expression arrays (Applied Biosystems) were used for *Il6* (Mm00446190_m1), *Ifn*γ (Mm99999071_m1), and *Ccl2* (Mm00441242_m1). Specific gene copy numbers were normalized to *Gapdh* (4352339E; Applied Biosystems).

Flow cytometry was performed as we have previously reported (31, 32). Whole lungs and the distal 15 cm of the small intestine were removed from selected mice and incubated in digest media for 45 min followed by the addition of EDTA (2 mM final concentration) for 15 min. Digest medium consisted of Dulbecco’s modified Eagle’s media supplemented with 5% fetal calf serum, sodium bicarbonate, penicillin/streptokinase, 10 mM Hepes, 250 U/mL collagenase I (Worthington Biochemical), 50 U/mL DNase I (Worthington Biochemical), and 0.01% hyaluronidase (Sigma-Aldrich, St. Louis, MO, USA) with or without 0.5 mg Brefeldin A (Sigma-Aldrich) in DMSO. The samples were filtered and the single cell suspension was treated with Red Blood Cell Lysing Buffer (Sigma-Aldrich) with or without Brefeldin A. APC-, FITC-, or PE-labeled antibodies against murine Mac-3/CD107b (clone M3/84), NK1.1 (clone PK 136), CD4 (clone RM4-5), CD8 (clone 53-6.7), CD3ε (clone 145-2C11), CD11b (clone M1/70), CD11c (clone N418), FcεRI (clone MAR-1), anti-mouse Lineage Cocktail (catalog #133305), and isotype controls IgGs (rat and Armenian hamster) were obtained from BioLegend (San Diego, CA, USA), eBioscience (San Diego, CA, USA), and/or BD Pharmingen (San Jose, CA, USA). For evaluation of depletion efficiency, anti-NK1.1 (clone 694370) and IgG2a control were obtained from R&D Systems (Minneapolis, MN, USA), and anti-CD4 (clone GK1.5) and IgG2b control (clone LTF-2) were obtained from BioXCell (West Lebanon, NH, USA). APC-labeled tetramer for the immunodominant epitope of SeV nucleoprotein (NP_324-332_) (35) was obtained from the NIH Tetramer Core Facility (Atlanta, GA, USA). Cells were treated with FC Block (ATCC, Manassas, VA, USA) stained with the above antibodies and analyzed on a FACSCalibur (BD Biosciences, San Jose, CA, USA).

For intracellular staining experiments, cells were isolated from mouse lungs in digest media supplemented with 20 mg/mL brefeldin A (Sigma-Aldrich). The single cell suspension was stained for surface markers as above in PBS/0.5% BSA supplemented with 20 mg/mL brefeldin A. Following this, the cells were washed and then incubated overnight at 4°C in 1% paraformaldehyde. The next morning cells were permeabilized with 50 µL Triton-X (MidSci, St. Louis, MO, USA) in 50 mL PBS/0.5% BSA. Cells were then stained with APC-labeled anti-mouse IFNγ antibody (clone XMG1.2), Foxp3 antibody (clone FJK-16s), or control APC-labeled anti-mouse IgG1κ (clone RTK 2071—for IFNγ) or IgG2aκ (clone eBM2a—for Foxp3) from eBioscience and/or BioLegend. Samples were analyzed on a FACSCalibur. Flow cytometry data were analyzed with Flow Jo software (Tree Star Inc., Ashland, OR, USA).

Bronchoalveolar lavage (BAL) cytokines were measured using an ELISA based array (Quansys Biosciences, Logan, UT, USA). After euthanizing mice, the pulmonary circulation was cleared of blood by injection of 1 mL sterile PBS into the right cardiac ventricle. The trachea was then cannulated with a 22-G catheter and BAL performed with 1 mL of sterile PBS. IL-1α, IL-1β, IL-2, IL-3, IL-4, IL-5, IL-6, IL-10, IL-12p70, IL-17, TNF, IFNγ, GMCSF, CCL2, CCL3, CCL5, CCL11, CCL17, CCL22, and CXCL1 were measured using an ELISA based array (Quansys Biosciences, Logan, UT, USA). The array was developed on the Li-Cor Odyssey according to the manufacturer’s instructions and analyzed with Q-View software (Quansys Biosciences, UT, USA).

### Adoptive Transfer of Regulatory T Cells (Tregs)

Splenocytes were isolated from naive *Foxp3^gfp^* mice and sorted into CD4^+^/Foxp3^+^ (Treg) and CD4^+^/Foxp3^−^ (effector T cells; Teff) based on scatter and CD4/GFP expression by flow cytometry. 10^6^ Treg or effector T cells were then injected i.p. into recipient C57BL6 mice that had been on RO water or RO water supplemented for 2 weeks with streptomycin. The mice were then inoculated with SeV and survival monitored.

### IFNγ Blockade and Cellular Depletion

On days 5 and 9 PI SeV, 0.1, 1, 10, or 100 µg of anti-mouse IFNγ antibody (clone XMG1.2) or control rat IgG1 antibody (BioLegend) were injected SQ. For NK1.1 depletion, anti-mouse NK1.1 antibody (clone PK136; BioXCell) or control mouse IgG2a (clone C1.18.4; BioXCell) was used, and for CD4 depletion anti-mouse CD4 (clone RM4-5; eBioscience) or control rat IgG2b (eBR2a; eBioscience) were used.

### Bacterial Genomic DNA Extraction, Quantitative PCR Amplification of 16S rRNA Gene Sequences, and 16S rDNA Sequencing

Lungs (lung parenchyma), trachea, and distal small intestine (distal 15 cm, DSI), cecum, and large intestine (LI) isolated from experimental animals were weighed and homogenized as described (19). Genomic DNA was extracted from tissues using the Qiagen stool kit as described (19). The abundance of specific bacterial groups was determined by qPCR using the MyiQ single-color real-time PCR detection system (Bio-Rad, Hercules, CA, USA) as described (19). Briefly, real-time PCR was done using the IQ SYBR Green Supermix (Bio-Rad), started with an initial step at 95°C for 3 min, followed by 40 cycles of 10 s at 95°C and 45 s at 63°C. Data were acquired in the final step at 63°C. Using the same genomic DNA from each sample, real-time PCR reactions were completed using group specific primers to determine the amount of bacteria in each of the following major groups: *Eubacterium rectale/Clostridium coccoides* (Erec), *Lactobacillus* sp. (Lact), *Bacteroides* sp. (Bac), Mouse Intestinal *Bacteroides*, Segmented Filamentous Bacteria (SFB), and total bacteria. Bacterial numbers were determined using standard curves constructed with reference bacterial plasmid standards containing the 16S sequence specific for each bacterial group analyzed. Data are presented as total burden per lung parenchyma or trachea, and bacterial burden per gram of intestinal tissue for the DSI, cecum, and LI.

Sequencing was performed by Diversigen™ (Baylor College of Medicine). The 16S rDNA V4 region amplicons (single index) were produced by PCR and sequenced on the MiSeq platform (Illumina) using the 2 × 250 bp protocol yielding pair-end reads that overlap by ~247 bp (36). Following sequencing, raw BCL files were retrieved from the MiSeq platform and called into fastqs by Casava v1.8.3 (Illumina). The read pairs were demultiplexed based on unique molecular barcodes, filtered for PhiX using Bowtie2 v2.2.1 (37), and reconstituted into two fastq files for each read using standard BASH. Sequencing Reads were merged (allowing 4 mismatches per ≥50 bases) and processed using USEARCH v7.0.1001 (38). Sequences were demultiplexed using QIIME v1.8.0 (39) and then clustered using the UPARSE pipeline (38). Operational taxonomic unit (OTU) classification was achieved by mapping the UPARSE OTU table to the SILVA database (40). Abundances were recovered by mapping the demultiplexed reads to the UPARSE OTUs. A custom script constructed an OTU table from the output files generated in the previous two steps.

### Bioinformatic Analysis

Microbiota data were analyzed using the Vegan 1.17-9 (41) and Ecodist 1.2.2 (42) packages in R 3.0.2 (43). Raw data, counts per genus-level OTU, were used to assess sample Simpson diversity. Microbiota data normalized to average sequencing depth were used to assess inter-sample Bray-Curtis beta diversity, sample clustering, and Non-metric Multidimensional Scaling Ordination (NMDS). Two antibiotic-treated lung samples failed sequencing and were excluded from subsequent data analysis. Statistical significance for differences in microbiota diversity between treatment groups was determined using Adonis (44). Normalized data were then log-transformed: Log_10_ (abundance + 1) for heteroscedastic Student’s *t*-test to find differences in microbiota abundance between treatment groups. *T*-test *p*-values were not corrected for multiple testing due to the relatively small number of tests performed, 30 for the cecum samples and 40 for the lung samples.

### Statistical Analysis

Survival data presented as Kaplan–Meier survival curves and statistical significance calculated by the Log-rank/Mantel–Cox test. Other data are presented as mean with statistical significance calculated by Student’s *t*-test or χ^2^ test (for dose–response to anti-IFNγ treatment). In all cases, *p* < 0.05 was considered statistically significant.

## Results

### Alteration of Intestinal Microbiota Increases Mortality From a Pulmonary Viral Infection

**Figure 1:**
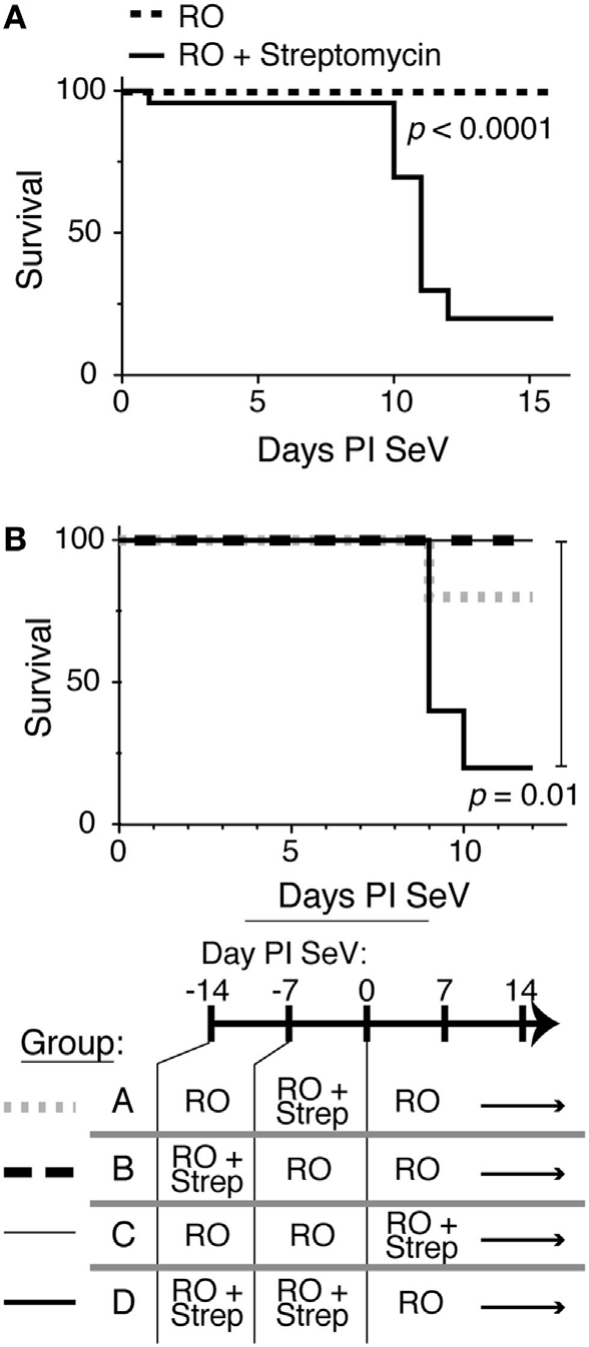
Disruption of intestinal microbiota results in increased mortality to respiratory Sendai virus (SeV) infection. **(A)** Kaplan–Meier survival curve for C57BL6 mice maintained on reverse osmosis (RO) water or RO water supplemented with 0.5 g/250 mL of the non-absorbable antibiotic streptomycin (RO + streptomycin) for 2 weeks prior to inoculation with 2 × 10^5^ plaque forming units SeV (RO, *n* = 26; RO + streptomycin, *n* = 23; data combined from six separate experiments, *n* ≥ 3 mice/treatment each). **(B)** Two weeks of RO + streptomycin water before the viral infection is sufficient to drive the increased mortality. Kaplan–Meier survival curve for mice exposed to RO or RO + streptomycin (RO + strep) water for the indicated time periods (*n* = 5/group).

To determine if intestinal microbiota could alter an immune response in the lungs, we took advantage of the antibiotic streptomycin and the well-characterized SeV infection model. Streptomycin is a non-absorbable antibiotic that has been shown only to affect bacterial load in the gastrointestinal tract with only 0.00001% being absorbed when given at high doses (45). Therefore, streptomycin treatment should have minimal to no effect on bacterial species in the lung. We placed C57BL6 mice on RO water or RO water supplemented with streptomycin. After 2 weeks, the mice were then infected with either SeV or UV-SeV. Normally, SeV infection leads to <5% mortality in infected mice (33). However, as shown in Figure 1A, mice that had been placed on streptomycin containing water had a marked and significant increase in mortality from SeV infection by days 10–12 PI with virus. This increase in mortality required an active viral infection, as mice inoculated with UV-SeV all survived regardless of antibiotic exposure.

Although streptomycin is not absorbed into the systemic circulation, it was still possible that the increased mortality resulted from a direct effect of the antibiotic on the antiviral immune response. However, as shown in Figure 1B, increased mortality was not seen when mice were given streptomycin for 1 week before (groups A and B) or just throughout the viral infection period (group C). In fact, the increase in mortality was seen only when 2 weeks of antibiotic therapy was given before viral infection (group D). This suggested that the effect of altering the intestinal microbiota required time to translate to the pulmonary mucosal immune response.

### Streptomycin Treatment Significantly Reduces Intestinal but Not Lung Microbiota Diversity

The protective and functional capacity of the microbiota depends on its composition, including species abundance and diversity. To determine the effect of streptomycin treatment on the richness of bacterial diversity, we used 16S high-throughput sequencing to examine the bacterial colonization of the cecum and lung.

**Figure 2:**
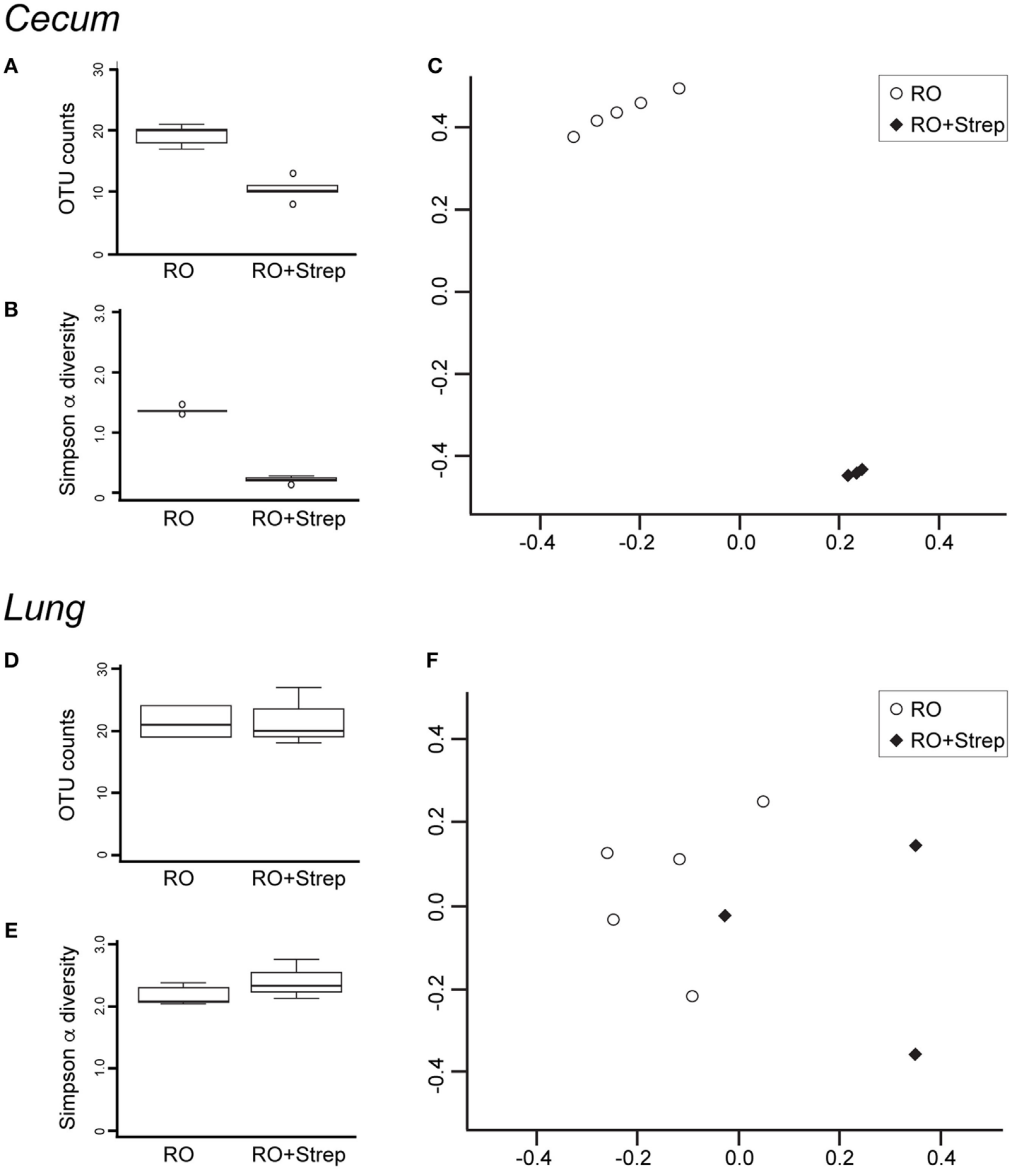
Streptomycin treatment reduces the abundance and diversity of cecal but not lung microbiota. DNA was isolated from cecum **(A–C)** and lung **(D–F)** after 2 weeks of reverse osmosis (RO) or RO + streptomycin treatment, and analyzed for microbial composition. Operational taxonomic unit (OTU) counts **(A,D)** and alpha diversity **(B,E)** were compared between treatment groups. Beta diversity was compared using Non-metric Multidimensional Scaling Ordination of the Bray–Curtis beta diversity metric **(C,F)**. **(A)** Significant differences were seen in cecal OTU count (*p* = 4.5 × 10^−5^, *n* = 5), **(B)** Simpson alpha diversity index (*p* = 1.5 × 10^−9^, *n* = 5), and **(C)** beta diversity (Adonis *p* = 0.012, *n* = 5, note that two treated samples were so close to the others that the treated group appears as only three samples). No significant differences were noted in lung microbiota **(D)** OTU composition, **(E)** Simpson alpha diversity index, or **(F)** beta diversity between antibiotic-treated (*n* = 3) and -untreated (*n* = 5) mice.

**Figure 3:**
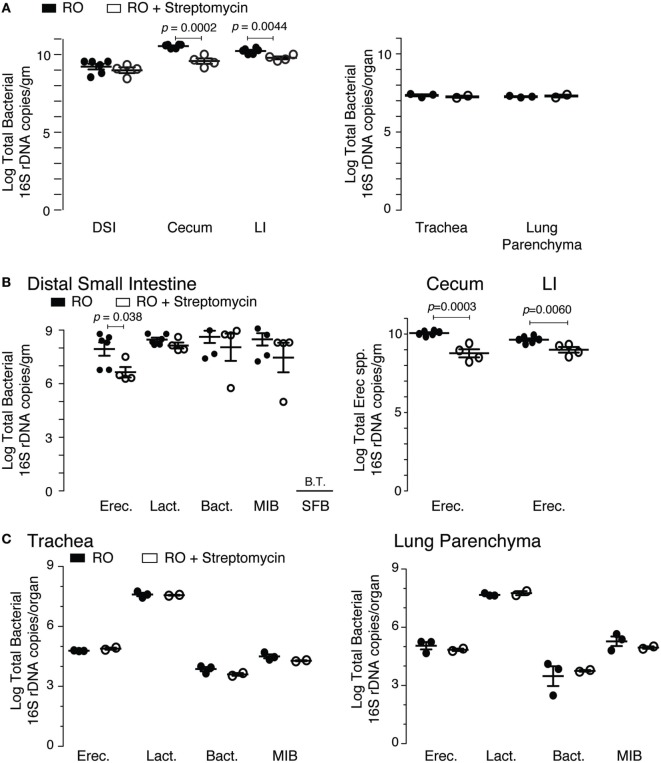
qPCR validation of streptomycin effect on intestinal and lung microbiota. **(A)** Total bacterial load (16S rDNA copies) in the intestinal and pulmonary compartments from mice exposed to 2 weeks of either reverse osmosis (RO) or RO + streptomycin water. Abbreviations: DSI, distal small intestine; LI, large intestine (*n* = 4–6 mice/group/tissue location). **(B)** Specific bacterial groups identified in the distal small intestine (left panel) and *Erec* spp. in the cecum and LI (right panel) from mice treated as in panel **(A)**. **(C)** Specific bacterial groups identified in the trachea (left) and lung parenchyma (right) from mice treated as in panel **(A)**. Microbiota determined by 16S qPCR, with quantities expressed as log total bacterial species per gram of intestinal sample or log total bacterial species per lungs or trachea (*n* = 2–6 mice/group/tissue location). Abbreviations: Erec, *Eubacterium rectale/Clostridium coccoides*, Lact, *Lactobacillus*, Bact, *Bacteroides*, MIB (S24-7), *Mouse intestinal Bacteroides*, SFB, *Segmented filamentous bacteria*, B.T., below threshold.

The microbiota composition of the cecum was predominantly *Clostridiales* (*Lachnospiraceae* and *Ruminococcaceae*), *Bacteroides*, and *Erysipelotrichales* (*Erysipelotrichaceae*). Antibiotic treatment significantly altered the microbiota in the cecum (Figure 2A). Detectable OTU count was reduced by 50% (*p* = 4.5 × 10^−5^), and the Simpson alpha diversity metric (Figure 2B) was reduced by 85% (*p* = 1.5 × 10^−9^). Most of the bacterial taxa were reduced to undetectable levels with the exception of *Bacillales* (*Paenibacillaceae* and *Planococcaceae*), which were relatively increased by 14,000 and 30-fold, respectively. Changes in microbiota abundance were validated by qPCR (Figure 3), with similarly characterized changes in the small and large intestines.

The microbiota composition in the lung was predominantly *Bacillales* (*Paenibacillaceae*) and *Clostridiales* (*Lachnospiraceae* and *Ruminococcaceae*). As expected with a non-absorbable antibiotic, streptomycin treatment did not induce significant changes in the lung microbiota. However, two treated lung samples failed sequencing and were excluded from subsequent data analysis. OTU count and Simpson diversity were comparable before and after treatment (Figures 2D,E). Analysis of the microbiota abundance suggested small changes in *Clostridiales* (*Lachnospiraceae*) and *Lactobacillales* (*Lactobacillaceae* and *Enterococcaceae*). However, analysis of the sequencing data revealed weak *p*-values (0.02, 0.02, and 0.04), which could not be validated by qPCR (Figure 3), suggesting that these changes were not significant. We, however, cannot completely rule out changes in specific low abundance species nor can we completely rule out a role for antibiotics in the failure of two of the treated lung samples.

Beta diversity analysis using the Bray–Curtis metric highlighted the dramatic change in composition induced by antibiotic treatment in the cecum. NMDS ordination showed control and treated cecum samples cluster distinctly (Adonis *p*-value = 0.012, Figure 2C), while the lung samples were intermixed (Figure 2F).

### Altering Intestinal Microbiota Leads to Dysregulated Pulmonary Immune Response to SeV

**Figure 4:**
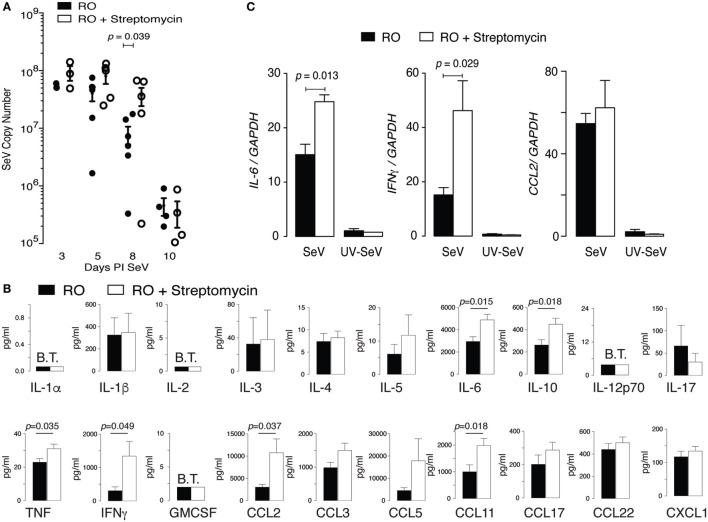
Altered intestinal microbiota augments pulmonary cytokine response to Sendai virus (SeV), but does not impair viral clearance. Mice were placed on reverse osmosis (RO) or RO + streptomycin water, and 2 weeks later inoculated with SeV. **(A)** RT polymerase chain reaction (PCR) quantification of SeV copy number in whole lungs at the indicated days post inoculation (PI) SeV (*n* = 3–6 mice/day and group). **(B)** Expression of cytokines in the bronchoalveolar lavage fluid of mice 8 days PI SeV. Data are array results from a commercially available ELISA protein array (data combined from five separate experiments with *n* ≥ 2/group/experiment, with samples run in duplicate); note increased IL-6, IFNγ, and CCL2 (MCP-1) protein. **(C)** Message for *Il6* and *Ifnγ*, but not *Ccl2*, was increased in whole lung of SeV-infected mice that had received streptomycin. Specific cytokine mRNA levels were assessed by RT PCR in whole of lung of mice at day 8 PI SeV or UV inactivated SeV (*n* = 3/group and treatment) and normalized to *Gapdh* copy number.

One possible cause for increased mortality in mice exposed to streptomycin before viral infection would be a failure to adequately control and clear the virus. To assess this possibility, we examined viral titers by qPCR. In wild-type C57BL6 mice, SeV titers peak around day 3–5 PI and essentially clear by day 12 PI (33). As can be seen in Figure 4A, treatment with streptomycin for 2 weeks before viral inoculation led to a small but significant decrease in viral clearance at day 8 PI. However, by day 10 PI, viral titers were identical regardless of treatment. Since the increased mortality was seen at day 10–12 PI, the half-log difference seen at day 8 PI is not sufficient to explain the increased death.

We next questioned whether the cytokine response was dysregulated in SeV infected streptomycin-treated mice. Using BAL fluid obtained from mice 8 days PI SeV, we performed a protein microarray (Figure 4B). Of the 20 cytokines and chemokines assayed, 6 were statistically increased with streptomycin treatment. Of these, the most highly expressed (i.e., in ng/mL concentrations) were IL-6, IFNγ, CCL2 (monocyte chemoattractant protein-1, MCP-1), and CCL11 (eotaxin). While CCL11 levels doubled with streptomycin exposure, they were unlikely to play a major role in the immune response, since SeV infection in C57BL6 mice is not associated with lung or BAL eosinophilia (primary target of CCL11). Of the other cytokines, IFNγ production was most increased with streptomycin exposure (4.4 versus 3.6-fold for CCL2 and 1.6-fold for IL-6), suggesting that it might be responsible for the increased mortality.

We next determined if the increase in pro-inflammatory cytokines required a live viral infection. Examining whole lung at day 8 PI SeV or UV-SeV we found streptomycin treatment led to a statistically significant augmentation in *Il6* and *Ifnγ* mRNA in infected mice, while uninfected mice had no such increase in these pro-inflammatory gene products (Figure 4C). *Ccl2* mRNA was unaffected by streptomycin treatment, although it was clearly induced in a viral-specific fashion. Since streptomycin treatment increased CCL2 protein without a concomitant increase in mRNA levels, CCL2 production likely is controlled in a post-translational fashion. The augmentation in both IL-6 and IFNγ production, on the other hand, appeared to be transcriptionally regulated as both protein and mRNA were induced with streptomycin treatment.

### Increased Mortality Is due to Overproduction of IFNγ

**Figure 5:**
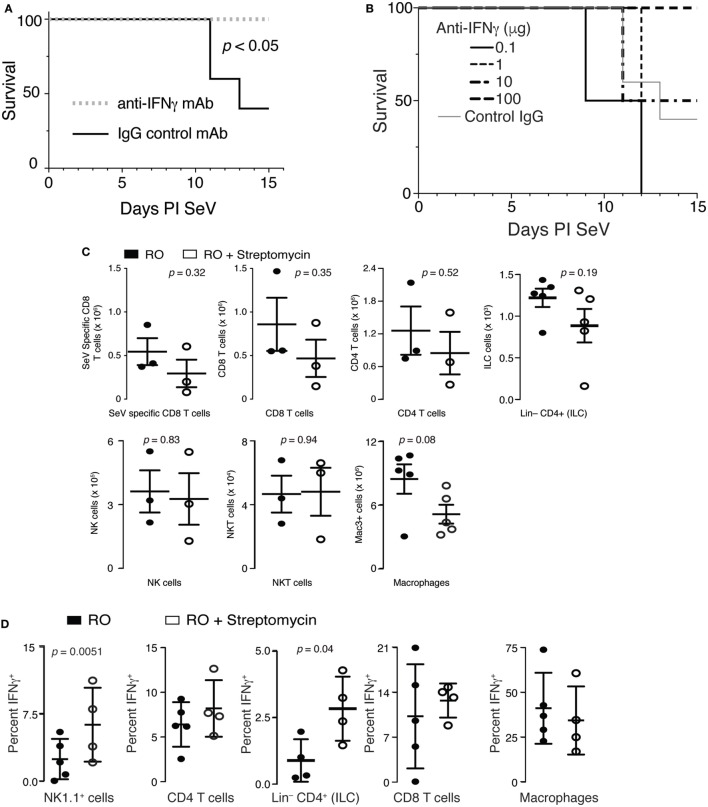
Increased mortality in streptomycin-treated mice is IFNγ dependent. **(A)** Mice were given streptomycin for 2 weeks before infection with Sendai virus (SeV). At day 5 and 9 PI SeV, 100 µg of an IFNγ blocking or control IgG monoclonal antibody was given SQ and mortality measured (*n* = 5/group). **(B)** Mice were treated as in panel **(A)** but with the indicated doses of anti-IFNγ antibodies being administered (*n* = 5/group). *p* < 0.04 for dose–response (χ^2^). **(C)** No difference was noted in the number of SeV-specific CD8^+^ T cells, total CD8^+^ T cells, total CD4^+^ T cells, NK cells (CD3^−^NK1.1^+^ lymphocytes), NKT cells (CD3^+^NK1.1^+^ lymphocytes), innate lymphoid cells (Lin^−^ CD4^+^ lymphocytes), and macrophages (Mac3^+^) at 8 days PI SeV in the lungs of mice treated with reverse osmosis (RO) or RO + streptomycin (*n* = 3–5 mice/group). **(D)** IFNγ from NK1.1, CD4, CD8-expressing lymphocytes, CD4^+^Lin^−^ cells, or lung Mac3^+^ macrophages was determined by intracellular flow cytometry on day 8 PI SeV. Mean ± SEM percent of given cell type expressing IFNγ is shown (*n* = 4–10 mice/treatment/group). See Figure S1 in Supplementary Material for gating strategy.

Overproduction of IFNγ during an immune response is well known to be fatal (46). Therefore, we hypothesized that increased production of IFNγ could be contributing to the excess mortality in mice that had ingested streptomycin prior to viral inoculation. To test this hypothesis, we placed mice on RO + streptomycin for 2 weeks before infecting them with SeV. On day 5 and 9 PI SeV, mice were given either an anti-IFNγ mAb or IgG control mAb SQ and mortality monitored. As demonstrated in Figure 5A, injection with an anti-IFNγ mAb completely prevented mortality to SeV infection. In fact, using decreasing doses of anti-IFNγ mAb, we were able to show a statistically significant dose–response in SeV-induced mortality (Figure 5B). Thus, the increased mortality from SeV in mice treated with streptomycin appears to be IFNγ dependent.

T cells, NK cells, NKT cells, innate lymphoid cells (ILCs)—including lymphoid tissue inducer (LTi), a subclass of ILCs, and macrophages are all cell types known to produce IFNγ. Therefore, to determine if streptomycin exposure altered the adaptive immune response to SeV, we examined whether lung T cell numbers were changed in mice treated with the antibiotic. Although SeV-specific (SeV tetramer^+^) CD8^+^ and total CD8^+^ and CD4^+^ T cells appeared to be reduced with streptomycin ingestion, none of these changes were significant (Figure 5C). Similarly, no difference was seen in the NK, NKT, or ILC cell compartments, although total lung macrophage numbers were modestly decreased. Together, these data demonstrate that increased IFNγ did not come from an increase in the number of IFNγ-producing cells.

Given no differences in cell numbers, we hypothesized that per-cell IFNγ production might be altered in streptomycin-treated and SeV-infected mice. Mice were placed on either RO or RO + streptomycin water for 2 weeks before being infected with SeV. At day 8 PI, lung cells were isolated and expression of IFNγ determined using intracellular flow cytometry. There was a significant and marked increase in the percent of NK1.1-expressing lymphocytes and Lin^−^CD4^+^ cells that were producing IFNγ when mice were treated with streptomycin before SeV infection, whereas the frequency of CD4^+^ or CD8^+^ lymphocytes, or lung macrophages producing IFNγ was not changed with streptomycin exposure (Figure 5D). Thus, NK1.1^+^ lymphocytes and/or Lin^−^CD4^+^ cells could be a source of the increased production of IFNγ found in streptomycin-treated, SeV-infected mice.

### Disruption of the Intestinal Microbiota Induced Mortality to SeV Is CD4 Not NK1.1 Dependent

**Figure 6:**
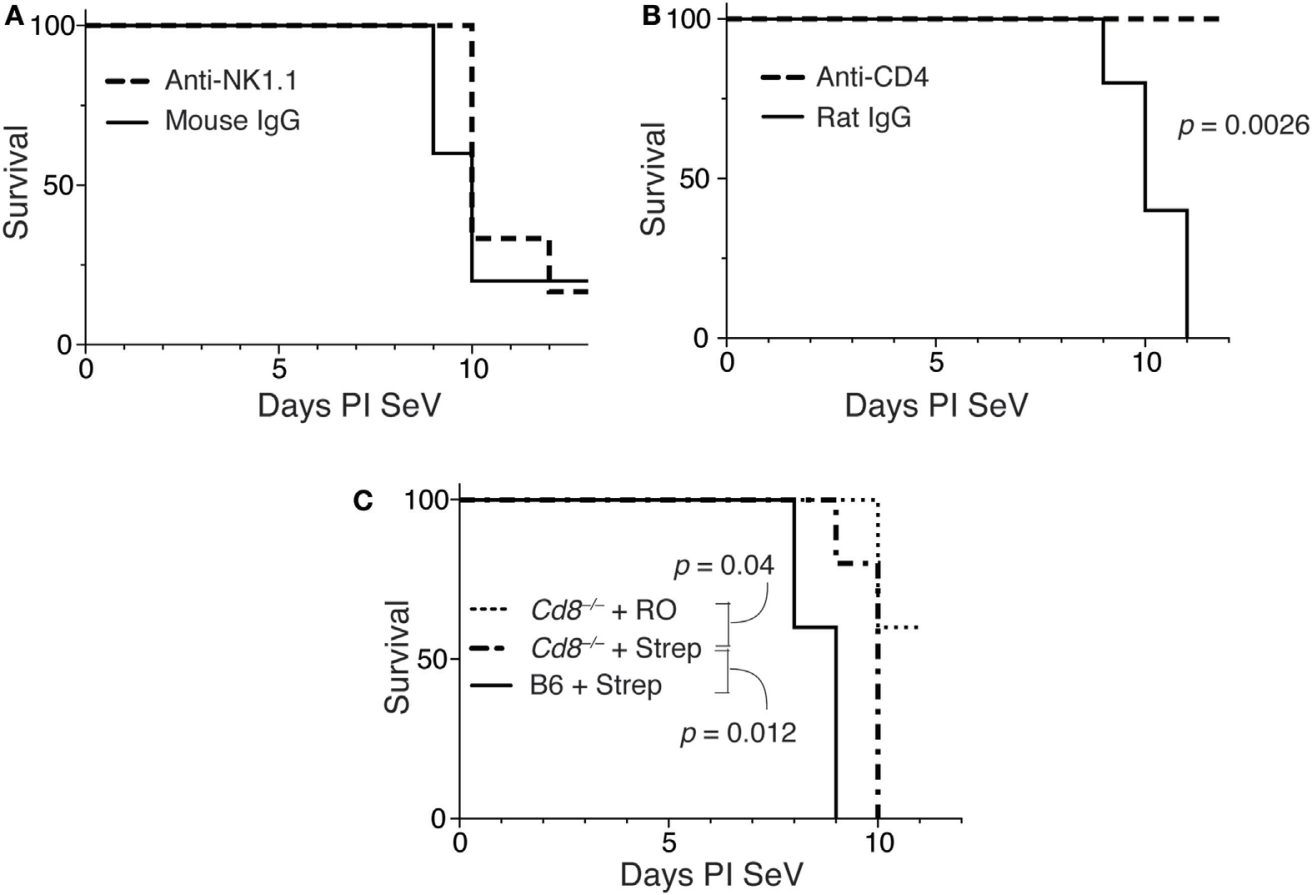
Increased mortality in streptomycin-treated mice requires CD4^+^ cells, but not IFNγ-producing CD4 T cells. **(A)** Mice were treated as in Figure 4, and at day 5 and 9 PI Sendai virus (SeV), 100 µg of anti-NK1.1 or control mouse IgG monoclonal antibody was administered SQ. Mortality was then measured. **(B)** Mice were treated as in panel **(A)** using anti-CD4 or control rat IgG monoclonal antibodies, and mortality was measured. **(C)** Wild-type (C57BL/6) or CD8-deficient (*Cd8^−/−^*) mice were treated as in panel **(A)**, and mortality measured. For all of these experiments *n* = 5/group/treatment.

To determine if NK1.1^+^ cells were necessary for the increased mortality, we gave mice RO or RO + streptomycin for 2 weeks before inoculating with SeV. At days 5 and 9 PI SeV, anti-NK1.1 or a control mAb was administered (see Figure S2A in Supplementary Material for depletion efficiency) and mortality measured. As shown in Figure 6A, depletion of NK1.1-expressing cells had no effect on the mortality to SeV.

To determine if CD4-expressing cells were necessary for the increased mortality, we examined the effect of depleting CD4 cells in this model (see Figure S2B in Supplementary Material). As shown in Figure 6B, when mice (on RO + streptomycin water) were given an anti-CD4 monoclonal antibody on days 5 and 9 PI SeV, they had no mortality, while mice given a control rat IgG still exhibited increased mortality. This demonstrates a requirement for a CD4-expressing cell. The major CD4-expressing cell types are CD4^+^ T cells, macrophages, NKT cells, ILCs (ILC1s), and LTi cells (47).

We next assessed the requirements of CD8-expressing T cells in the model. As shown in Figure 6C mice deficient in CD8 (*Cd8^−/−^*) demonstrated significant mortality with SeV even when only on RO water. However, mortality was further significantly increased with dysregulation of intestinal microbiota, although there was modest (but significant delay) in mortality when compared with wild-type (C57BL/6) mice. Together, this suggests minimal requirement for CD8^+^ T cells in the increased mortality phenotype.

Thus, increased mortality appeared to be dependent on IFNγ, and CD4 but not NK1.1 or CD8-expressing cells. Since only NK1.1^+^ cells and potential ILCs (Lin^−^ CD4^+^ cells) were shown to have increased IFNγ production, we presume that ILCs are the likely source of the IFNγ. We are unable to verify ILCs as the source of IFNγ because the currently available ILC-deficient mice (i.e., *Rag*γ*c^−/−^*) also lack T and B cells, and, thus, would rapidly succumb to SeV infection (48). However, as mentioned, the lack of an increase in IFNγ from CD4^+^ T cells and macrophages, as well as the fact that the anti-NK1.1 treatment did not prevent mortality, suggests that the increased IFNγ came from the lineage negative population.

### Streptomycin Reduces Tregs, Which Are Required to Prevent Mortality

**Figure 7:**
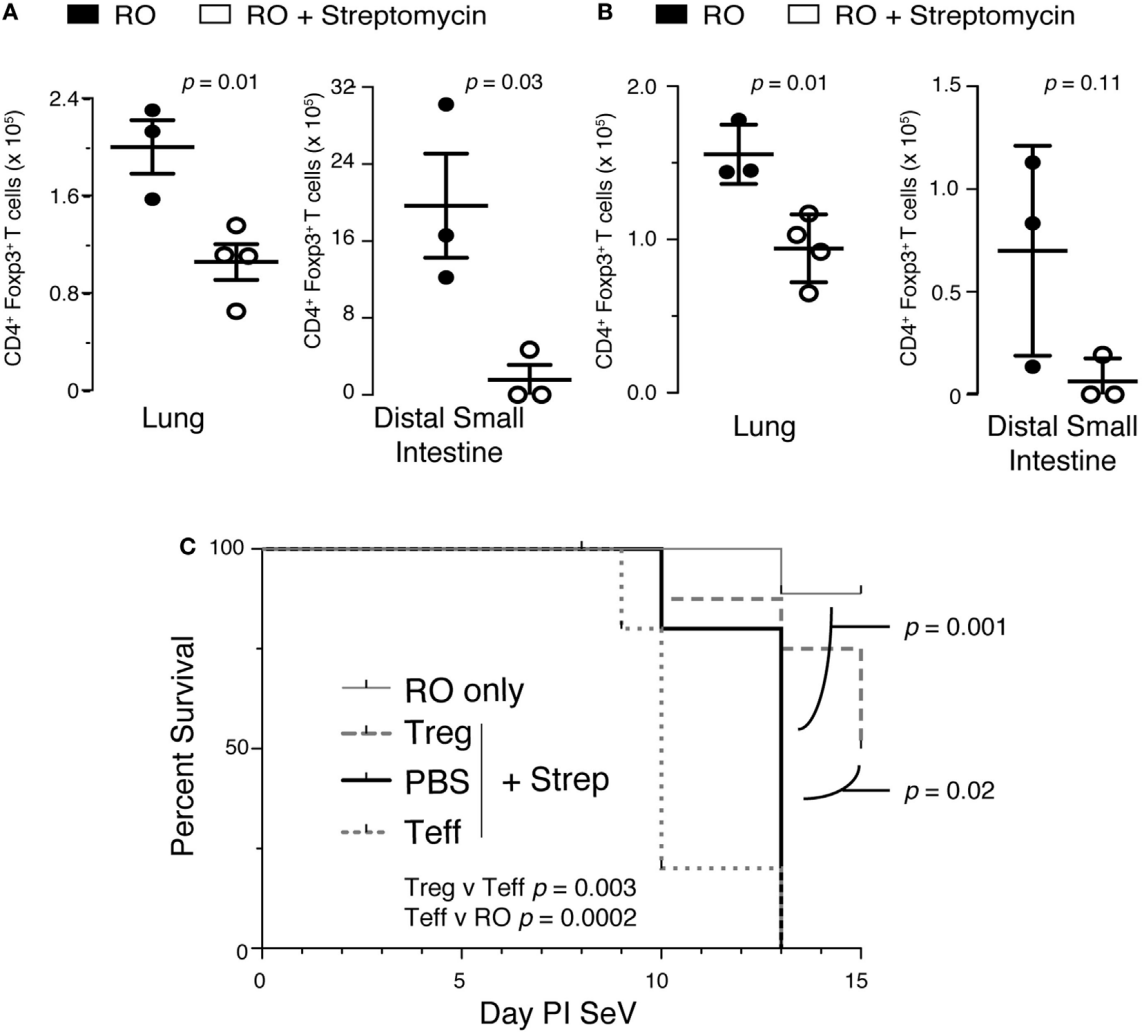
Disruption of intestinal microbiota reduces lung and intestinal regulatory T cells (Tregs), which are needed to prevent increased mortality to Sendai virus (SeV). **(A)** Foxp3^+^ Tregs isolated from the lung or distal small intestine from mice treated with 2 weeks of reverse osmosis (RO) or RO + streptomycin (*n* = 3–5 mice/group). **(B)** Mice treated as in panel **(A)** but after 2 weeks of RO or RO + streptomycin water the mice were infected with SeV and number of Foxp3^+^ Tregs determined at day 8 PI SeV (*n* = 3–4 mice/group). **(C)** Adoptive transfer of CD4^+^/Foxp3^+^ Treg but not CD4^+^/Foxp3^−^ effector T (Teff) cells at the time of SeV infection prevents the increased mortality seen in mice given 2 weeks of streptomycin before viral infection. RO only = mice given RO water and infected with SeV without any transfer of cells; Treg + Strep, PBS + Strep, and Teff + Strep are mice given 2 weeks of streptomycin followed by adoptive transfer of the given cells and SeV inoculation (*n* ≥ 5 mice/group, data combined from two separate experiments).

We next hypothesized that alteration in intestinal microbiota might lead to disruption of a regulatory component in the lung. One controller of the adaptive immune response is the Treg, so we examined the effect of streptomycin treatment on Treg numbers in the lung and gut. Surprisingly, Tregs were significantly reduced in the lung parenchyma with streptomycin treatment (Figure 7A). In addition, antibiotic treatment led to an almost complete absence of *Foxp3^+^* Tregs in the distal small intestine. This effect was sustained in the lung during infection with SeV (Figure 7B). In the gut, SeV infection reduced Treg numbers in both RO and RO + streptomycin-treated animals (compare Figures 7A,B); however, the difference between the two treatments was not significant with SeV infection (*p* = 0.11). At present, we are unable to explain the reason why SeV infection shows a lower trend for Tregs numbers even without antibiotic treatment. To determine if loss of Tregs was responsible for the increased mortality, we performed adoptive transfer experiments. Mice were kept on streptomycin or RO water for 2 weeks then given 1 × 10^6^ splenic Tregs *via* i.p. injection and then inoculated i.n. with SeV the same day and mortality monitored over time. We have used Tregs derived from splenocytes because they are routinely used in adoptive transfer studies and have shown to resolve lung inflammation, injury, and lethality due to Treg deficiency (49–51). Transferring Treg but not effector CD4^+^ T cells (Teff) significantly reduced the viral induced mortality in SeV-infected mice (Figure 7C). Interestingly, while not statistically significant, there was a trend (*p* = 0.064) for *increased* mortality with transfer of Teff cells, supporting our CD4 depletion data (Figure 6B). It is worth noting that ILCs would have been included in our Teff cell population (since they express CD4) and could have led to the trend for increased mortality with Teff transfer. Taken together, these data suggest that modulating the intestinal microbiota before a respiratory viral infection will drive a dysregulated immune response characterized by both a reduction in lung Tregs and an increase in IFNγ production that together lead to increased mortality.

## Discussion

We are only beginning to understand the importance of commensal microbiota in modulating host immune responses. Most prior studies have focused on the *intestinal* microbiota and *intestinal* immune response; for example, the presence of SFB was associated with the development of Th17 cells (2–4, 52), and intestinal microbiota appear critical for *intestinal* Treg development (7–9). There is evidence that intestinal microbiota influences systemic disease development, including obesity (12), non-alcoholic fatty liver disease (53), experimental allergic encephalomyelitis (5), and autoimmune arthritis (6). However, few studies have been related to the lung, and most focused on disease correlation not immune mechanism (17). Nonetheless, several studies have shown a potential effect of reduced microbiota diversity in development of asthma and atopic disease (15, 30, 54).

Our data strongly suggest that changes in the intestinal microbiota are sufficient to alter the immune response to a pulmonary viral infection. This alteration led to increased production of inflammatory cytokines with markedly increased mortality. Our data suggest that during homeostasis, intestinal microbiota condition the respiratory immune system so that during a viral infection production of pro-inflammatory cytokines, like IFNγ, is regulated. With the response regulated, mice survive. However, alteration of the intestinal microbiota reduces the regulatory oversight, allowing for increased IFNγ production, leading to increased mortality from the infection.

There are some caveats to our study. First, while streptomycin is thought to be a non-absorbed antibiotic, it is still possible that some of the antibiotic did reach the lung. However, any amount that reached the lung was too small to significantly alter the lung microbiota. Furthermore, it is important to note even with this lack of a significant change in lung microbiota it is still possible that minor effects on lung microbiota could have affected the outcome of our studies.

Our antibody depletion data showed that a CD4-expressing cell was needed to drive the increased mortality. A prior study demonstrated increased expression of IFNγ in lung CD4^+^ T cells following influenza virus infection. These effector T cell were able to migrate to the intestine and modify the gut microbiome (55). However, we did not observe an increase in IFNγ in CD4^+^ T cells from SeV-infected lungs following streptomycin treatment. ILCs, and in particular the ILC1 and LTi subset, express CD4 and produce IFNγ (47, 56, 57). Therefore, our data support the idea that the CD4^+^ cells producing IFNγ were in fact an ILC subclass of cells. It is worth noting that depletion of CD4 cells would deplete both Tregs and the IFNγ-producing Lin^−^CD4^+^ cells. We believe the reduction in mortality is due to the fact that in the absence of the IFNγ-producing cells, the protective effect of the Tregs is unnecessary.

Our findings may be an extension of the concept of pathogen tolerance, which suggests that the severity of host response to infection is more dependent on host fitness than pathogen virulence, and the ability to tolerate specific pathogens is part of a host organism’s defense strategy (58, 59). While this concept focuses on host–pathogen interactions that induce tolerance, our model suggests that host fitness and tolerance to a viral pathogen infection is driven by intestinal commensal–host interaction. In our model, the gastrointestinal microbiota may interact with the immune system to modulate the pulmonary immune response, preventing an excessive and lethal inflammatory response to SeV. However, once the microbiota is disrupted and microbial diversity reduced, this modulation is lost. This triggers a loss of tolerance for the SeV infection, resulting in increased mortality.

Humans with the least diverse intestinal microbiota include infants and the elderly (60–62)—these are the same populations that have the highest rate of mortality to another *paramyxovirus*, RSV (63). However, whether reduced diversity contributes to increased mortality in humans infected with RSV remains to be examined. Interestingly, the *orthomyxovirus*, influenza, is not associated with an increased rate of mortality in infants (63), and an influenza mouse model showed no mortality effect when mice were treated with a non-absorbable antibiotic (29). This may indicate a differential effect of microbial diversity depending on the specific respiratory viral pathogen, or that alterations in specific microbial communities may be more critical to disease outcome than overall microbial diversity.

Recent evidence suggests that the use of antibiotics with different spectra of activity result in very different lung immune responses to influenza virus. For example, treatment with neomycin, a non-absorbable antibiotic, led to a modest decrease in viral clearance without any effect on mortality in a mouse model of influenza infection (29), whereas another report demonstrated that treatment with a cocktail of five antibiotics (ampicillin, gentamicin, metronidazole, neomycin, and vancomycin) not only reduced influenza virus clearance but also led to increased mortality to the virus (64). While the immune responses to influenza and *paramyxoviruses* (such as SeV and RSV) may well be disparate, it is also likely that the effects of the distinctive antibiotics on the intestinal microbiota were different. With the use of both absorbable and non-absorbable antibiotics, alterations in lung microbiota also could contribute to differences in host immune response. Our study used only streptomycin, a non-absorbable antibiotic that altered the intestinal microbiota without significantly affecting the lung microbiota, strongly supporting our conclusion that the host pulmonary immune response was being modulated by intestinal commensal–host interactions. While we cannot absolutely rule out any unknown direct toxic effect of the streptomycin, no known signs of streptomycin toxicity were observed in any of the mice. Furthermore, the doses used in this study were well below described toxic concentrations of streptomycin in mice (65).

The effect of streptomycin, albeit limited to the gastrointestinal tract, was broad, reducing both diversity and total bacterial burden. While this antibiotic, like other aminoglycosides, is most effective against aerobic bacteria, it still has effects against facultative and obligate anaerobes, as are found in the gastrointestinal tract and is commonly used to disrupt the gastrointestinal microbial ecology. It is important to note that changes seen in the intestinal microbiota may not directly reflect susceptibility to streptomycin, as disruption of gastrointestinal bacterial species often leads to complex outcomes due to alterations in bacterial–bacterial and bacterial–host interactions. Because of the broad changes in microbial composition, demonstrated by 16S sequencing, we were unable to identify any specific individual bacterial species that appeared responsible for the altered viral immune response. However, our studies demonstrate significantly decreased abundance of the EREC (*Clostridium*) bacterial group by qPCR. To the best of our knowledge, this is the first study linking reduction in lung Tregs to dysbiosis of gut microbiome; however, other reports have indicated that treatment with streptomycin can alter the pulmonary T-cell profile (66). Furthermore, effects of targeting indigenous bacterial species in the gut and colon is consistent with previously reported associations between *Clostridium* clusters IV, XIVa, and XVIII and Treg induction (7, 67, 68). Future studies will be needed to identify and characterize specific bacterial–bacterial and bacterial–host interactions in the intestinal tract that may be responsible for these immunologic outcomes.

Antibiotic disruption of the intestinal microbiota imparts a marked change on the pulmonary immune response to a respiratory paramyxoviral infection, as manifested by our work. While these data clearly show a role for IFNγ in the mortality to an otherwise nonlethal viral infection, the direct contribution of Tregs is less clear. The reduction in mortality with Treg transfer could be due to the Tregs, but very possibly could be through mechanisms not directly related to their regulatory functions. This was something that our current study does not address. It is also worth noting that although currently there is no study showing direct migration of Tregs from gut to lung, there is evidence that short chain fatty acids (acetates) from the gut could regulate Tregs in the lungs (69, 70). These data highlight the connection between the intestinal microbiota and the pulmonary immune system and raise concerns over the potential risk of indiscriminate antibiotic use. While the overall impact of these findings on disease awaits further studies, we have begun to examine more fully the bacterial species involved in this response and the specific components of the pulmonary and intestinal immune systems that are most responsive to the microbiota. Clearly the intestinal microbiota plays a significant role in the immune response to pathogens at distant mucosal surfaces. Future studies will continue to provide insight into the importance of these symbionts to health and disease.

## Ethics Statement

This study was carried out in accordance with the recommendations of the Institutional Animal Care and Use Committees of the Medical College of Wisconsin and the Research Institute at Nationwide Children’s Hospital. The protocol was approved by the Institutional Animal Care and Use Committees of the Medical College of Wisconsin and the Research Institute at Nationwide Children’s Hospital.

## Author Contributions

MG, LC, S-RH, DC, and NS designed the experiments, analyzed the data, prepared the figures, and wrote and corrected the manuscript. LC, S-RH, SZ, MH, DH, JS, and MR performed the experiments and assisted in analyzing the results. VL analyzed the data, prepared the figures, and corrected the manuscript. All the authors reviewed the results and approved the final version of the manuscript.

## Conflict of Interest Statement

MG has received research support from Polyphor. The remaining authors declare that the research was conducted in the absence of any commercial or financial relationships that could be construed as a potential conflict of interest. The reviewer SR and handling Editor declared their shared affiliation.

## Supplementary Material

Figure S1 Gating strategy for flow cytometry. Single cell suspension of lung cells was analyzed by flow cytometry as shown. **(A)** Macrophages were identified as large side scatter (SSC) cells that were Mac-3^+^, while lymphocytes were identified by their SSC and forward scatter (FSC), and then were evaluated for expression of CD4, CD8, CD3e, NK1.1, and SeV tetramer as shown. **(B)** Lymphocytes were identified as in panel **(A)** and then CD4^+^ lineage cocktail—(Lin^−^) cells were examined by intracellular flow cytometry for expression of IFNγ (compared with isotype control). Representative data shown.

Figure S2 Depletion of NK1.1 and CD4 in mouse lung. **(A)** Efficiency of NK1.1 depletion. Representative histograms of lung cells stained for NK1.1 (clone 694370) from mice who had received 100 µg IgG2a isotype control (clone C1.18.4) or anti-NK1.1 (clone PK136) i.p. 5 days previously. Bar graph shows frequency of NK1.1-expressing cells in the lymphocyte gate with each treatment. Treatment with anti-NK1.1 led to an average of 97% reduction of NK1.1-expressing cells compared with the IgG control. **(B)** Similar experiment as in panel **(A)** but following CD4 depletion (100 µg clone RM4-5 or eBR2a) and stained with anti-CD4 (clone GK1.5) or isotype control (IgG, clone LTF-2). CD4^+^ lymphocytes were reduced by 98.3% at day 5 compared with IgG control. **p* < 0.05, *n* = 2/treatment.
