# Supplementary figures and images for: Intestinal Microbiota Disruption Reduces Regulatory T Cells and Increases Respiratory Viral Infection Mortality Through Increased IFNγ Production
Source: Front Immunol. 2018 Jul 10;9:1587. doi: 10.3389/fimmu.2018.01587 (PMC6048222; doi:10.3389/fimmu.2018.01587)

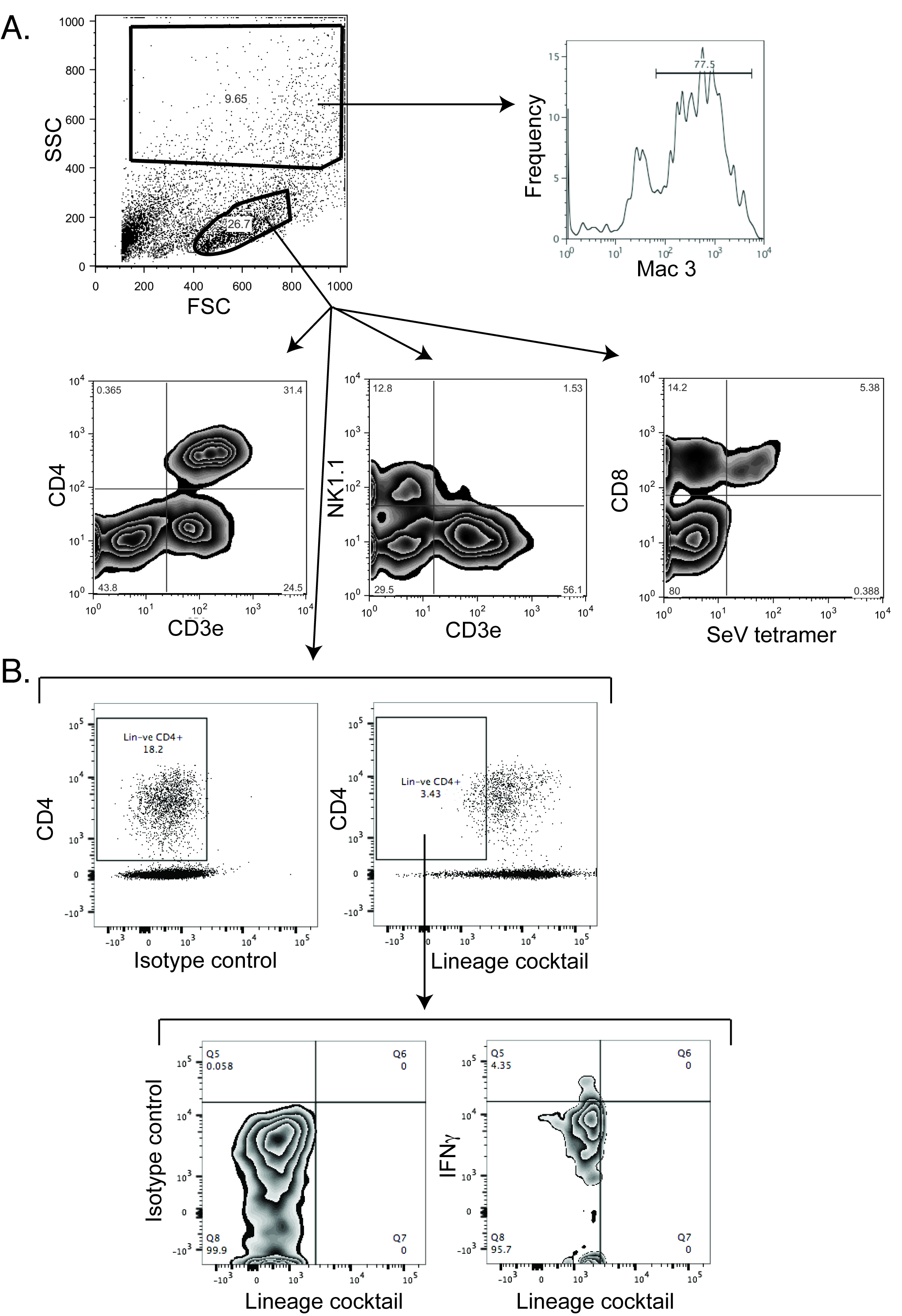

Supplement: Figure S1 — Gating strategy for flow cytometry. Single cell suspension of lung cells was analyzed by flow cytometry as shown. (A) Macrophages were identified as large side scatter (SSC) cells that were Mac-3+, while lymphocytes were identified by their SSC and forward scatter (FSC), and then were evaluated for expression of CD4, CD8, CD3e, NK1.1, and SeV tetramer as shown. (B) Lymphocytes were identified as in panel (A) and then CD4+ lineage cocktail—(Lin−) cells were examined by intracellular flow cytometry for expression of IFNγ (compared with isotype control). Representative data shown. [file Image_1.tif]

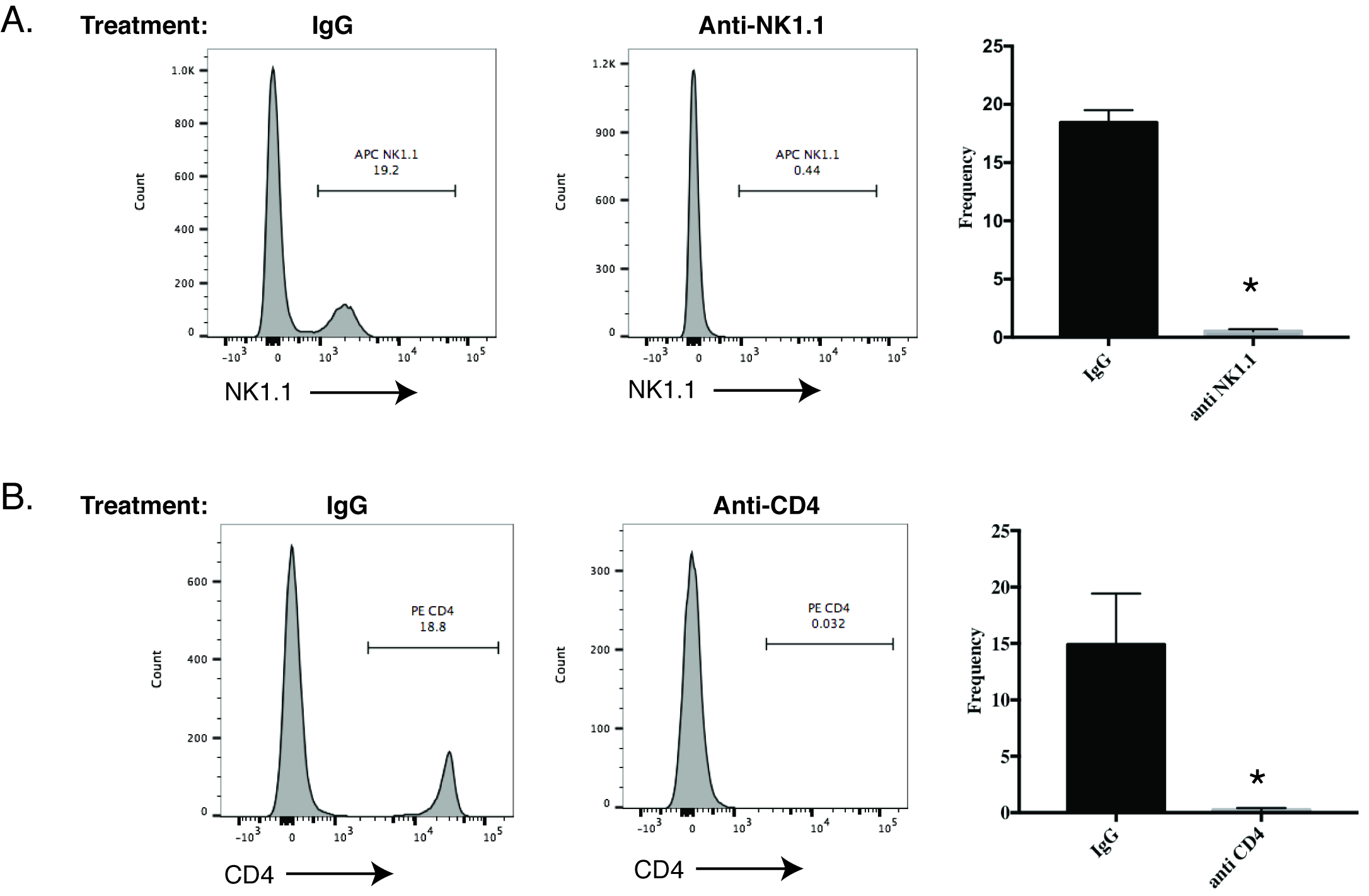

Supplement: Figure S2 — Depletion of NK1.1 and CD4 in mouse lung. (A) Efficiency of NK1.1 depletion. Representative histograms of lung cells stained for NK1.1 (clone 694370) from mice who had received 100 µg IgG2a isotype control (clone C1.18.4) or anti-NK1.1 (clone PK136) i.p. 5 days previously. Bar graph shows frequency of NK1.1-expressing cells in the lymphocyte gate with each treatment. Treatment with anti-NK1.1 led to an average of 97% reduction of NK1.1-expressing cells compared with the IgG control. (B) Similar experiment as in panel (A) but following CD4 depletion (100 µg clone RM4-5 or eBR2a) and stained with anti-CD4 (clone GK1.5) or isotype control (IgG, clone LTF-2). CD4+ lymphocytes were reduced by 98.3% at day 5 compared with IgG control. *p < 0.05, n = 2/treatment. [file Image_2.tif]
